# From catastrophizing to catalyzing: does pain catastrophizing modulate the beneficial impact of open-label placebos for chronic low back pain? A secondary analysis

**DOI:** 10.3389/fpsyg.2025.1522634

**Published:** 2025-06-25

**Authors:** Elif Buse Caliskan, Katharina Schmidt, Andreas Hellmann, Tamás Spisák, Ulrike Bingel, Julian Kleine-Borgmann

**Affiliations:** ^1^Department of Neurology, Center for Translational and Neuro- and Behavioral Sciences, University of Duisburg-Essen, Essen, Germany; ^2^Department of Anesthesiology, University of Duisburg-Essen, Essen, Germany; ^3^Center for Translational Neuro- and Behavioral Sciences, University of Duisburg-Essen, Essen, Germany

**Keywords:** back pain, placebos, placebo effect, catastrophization, treatment outcome

## Abstract

Chronic back pain (CBP) is a global health problem with significant health and economic consequences. Traditional analgesics are often no better than placebo, highlighting the need for biopsychosocial approaches. Open-label placebos (OLPs), administered with patient consent, offer a promising alternative. Existing research has mainly focused on the effects of OLP treatments on patient-reported outcomes. In a previous randomized controlled trial (RCT), we investigated whether an OLP treatment improves subjective and objective outcomes such as spinal mobility in CBP patients. The analysis showed significant reductions in pain intensity, disability, and depressive symptoms after OLP combined with treatment-as-usual (TAU). However, objective improvements in spinal mobility were not observed. In this exploratory analysis, we aimed to identify predictors of objective improvement after OLP treatment. Psychological factors (e.g., depression, stress, and pain catastrophizing) and baseline physiological measures were analyzed using generalized linear models. Results showed that patients with lower pain catastrophizing exhibited increased spinal motion velocity in the OLP+TAU group, while those with higher pain catastrophizing did not. These findings suggest that OLP treatment may provide measurable benefits for a specific subset of patients, supporting its potential as a personalized intervention in managing CBP. Further research is needed to confirm these findings and to elucidate the role of psychological factors in chronic pain management.

## Introduction

Chronic back pain (CBP) is defined as back pain that persists for more than 12 weeks even after treating the underlying cause or injury, develops in ~5–10% of all cases of back pain, and represents one of the most common and recurrent musculoskeletal pain conditions (Ehde et al., 2014). Approximately 9% of the adult population worldwide suffers from CBP, with prevalence increasing with age (Meucci et al., 2015). Worldwide, CBP is the second leading cause of disability and represents a significant health and economic burden (Ferreira et al., 2023). Treatment of CBP often requires a multidisciplinary approach that incorporates the biopsychosocial model of pain, as this condition frequently responds poorly to monotherapy with analgesics (Ehde et al., 2014). In line with this, several commonly prescribed therapies for CBP have been shown to be either marginally or no more effective than placebo controls in double-blind, randomized clinical trials (Machado et al., 2015; van der Gaag et al., 2020), implying that placebo responses in these RCTs may be substantial, and go beyond natural fluctuation of symptoms or regression to the mean (Kaptchuk and Miller, 2015; Skyt and Vase, 2016).

Placebo effects refer to the improvement of health outcomes resulting from a patient's involvement in the therapeutic experience rather than the treatment itself. How patients perceive the surrounding psychosocial treatment context induces placebo effects through expectations and conditioning. By shaping a conceptual image of upcoming sensory events, expectations can significantly influence the neural processes of perceiving actual sensory stimuli (Petrie and Rief, 2019). Moreover, unconscious conditioning happens when contextual cues (e.g., taste, shape, color of the pill, or the decoration of the therapeutic milieu) serve as conditioned stimuli. These cues can elicit clinical improvements after being repeatedly paired with an unconditioned stimulus, like an active drug (Jensen et al., 2015). However, leveraging placebo effects in treatment settings (e.g., through placebo pills) presents an ethical challenge for clinicians, as traditional practice assumes that deception is required for placebos to produce beneficial effects. This involves misleading patients by presenting fake pills as actual medication or prescribing active medication with no known efficacy for the underlying condition (e.g., vitamin supplements for pain), violating patients' autonomy and the concept of informed consent (Enck et al., 2013).

In response to these concerns, open-label placebos (OLPs) have emerged as a promising alternative to harness placebo effects in clinical settings. Administered openly to patients, OLPs can induce placebo effects transparently, without deception, and offer a viable and ethical option for harnessing placebo effects for the treatment of chronic and functional conditions (Blease et al., 2020). Indeed, emerging evidence supports the efficacy of OLPs in various clinical conditions, particularly for episodic (i.e., migraine) or chronic pain (i.e., irritable bowel syndrome, chronic back pain). In a large, randomized treatment-as-usual (TAU) controlled trial with *N* = 122 patients suffering from CBP, we demonstrated the efficacy of a 3-week OLP treatment as an add-on to TAU (Kleine-Borgmann et al., 2019). After 3 weeks, OLPs led to a significant reduction in pain intensity (*d* = 0.44), subjective disability (*d* = 0.45), and depressive symptoms (*d* = 0.5). Noteworthy, the OLP-associated improvement in back pain was comparable with the reported efficacy of a commonly prescribed analgesic, i.e., etoricoxib (*d* = 0.32; Birbara et al., 2003). Within the group treated with OLPs, pain reduction and improved subjective disability persisted for up to 3 months of follow-up. Interestingly, there was no OLP-associated change in objective parameters, such as range or velocity of spine motion. These results were confirmed in a meta-analysis by von Wernsdorff et al. (2021), who reported an overall significant effect of OLPs compared with no treatment in 11 clinical trials involving patients suffering from migraine, back pain, allergic rhinitis, cancer-related fatigue, menopausal hot flashes, and attention-deficit hyperactivity disorder.

However, the mechanisms underlying OLP efficacy across different conditions are not fully understood. A growing body of evidence recently highlighted the importance of expectations (Buergler et al., 2023) and distinguished shared as well as distinct neurobiological underpinnings of OLPs and “traditional” placebos (Benedetti et al., 2023; Schaefer et al., 2022). Furthermore, it has been proposed that OLP may work through specific psychological traits, such as openness to experience, flexibility of thought, extraversion, hope, and curiosity, which contribute to how the patients cope with their symptoms (Haas et al., 2022; Kaptchuk et al., 2020). Importantly, research also suggests that placebo responses may differ between healthy participants and chronic pain patients due to variations in treatment expectations and underlying neurobiological mechanisms (Rossettini et al., 2023). In this exploratory analysis of our previously published trial showing OLP efficacy in CBP, we aim to identify psychological factors that contribute to the effect of a three-week OLP treatment on (semi-)objective outcomes, i.e., range and velocity of motion as well as measures of pain-related disability and quality of life. Given the limited prior research on psychological predictors of OLP efficacy in objective disability parameters, this study should be considered hypothesis-generating rather than hypothesis-confirming.

## Methods

### Design

This study is a secondary analysis of data acquired in a parallel-group randomized treatment-as-usual (TAU) controlled trial following a pretest-posttest design. The objective was to evaluate the impact of a 3-week OLP treatment in addition to TAU on pain intensity, wellbeing, functional disability, and objective spine movement for patients with CBP. The parent study also included an exploratory follow-up period of 3 months. A 3-year follow-up analysis of data collected in the original trial is also available but is not included in this secondary analysis (Kleine-Borgmann et al., 2023). Ethical and regulatory approvals were obtained from the local ethics committee of the Medical Faculty of the University of Duisburg Essen (16-7218-BO). The trial was conducted at the Essen Back Pain Center, University Hospital Essen, Essen, Germany, between March 27, 2017, and May 29, 2018, following the Declaration of Helsinki. The study design as well as the initial analysis plan were registered at the German Clinical Trials Center (Study ID DRKS00012712) on July 12, 2017 (updated on April 7, 2018) and were also described in detail in the publication of the primary findings (Kleine-Borgmann et al., 2019).

### Study population

A total of 122 patients suffering from CBP participated in the parent study. Patients were recruited either at the Essen Back Pain Center or through regional advertisements. The eligibility criteria included persistent back pain of a minimum duration of 12 weeks and age >18 years. Patients with any history of malignancy within the past 5 years, neurological deficits, any severe psychiatric disorder, or reporting a clinically non-significant pain (referred to as a mean pain intensity <4 on an 11-point numeric rating scale (NRS) a week before baseline) were excluded. The dose and frequency of established TAU at the time of enrolment had to remain stable for 3 weeks before screening and throughout the study. All patients gave written informed consent and received monetary compensation for their participation.

### Intervention

Before the informed consent procedure, standardized information about the placebo effect and the potential benefit of OLP was provided using a video, as reported and provided in the supplementary content of the initial publication (Kleine-Borgmann et al., 2019). Subsequently, all patients were randomly allocated in a 1:1 ratio to one of the experimental groups: (1) OLP in addition to TAU (*n* = 63, OLP+TAU group) and (2) TAU only (*n* = 59, TAU group). Due to the nature of OLP trials, patients were not blinded to the group allocation. To improve compliance and overcome possible negative effects arising from the allocation to the control group (e.g., disappointment), participants in the TAU group were offered the opportunity to receive OLPs upon the completion of the study. Patients in the OLP+TAU group received OLP capsules twice daily for 21 days in addition to their stable TAU. All OLP capsules were white gelatine capsules containing microcrystalline cellulose (Zeebo Effect^®^, LLC, South Burlington, Vermont, USA). Patients were instructed that the capsules contain no active ingredient. Capsule intake was documented daily in a patient diary. A blinded examiner carried out the assessments of outcome measures, and patients were asked to keep their group allocations confidential when in contact with blinded staff.

### Outcome measures

The primary outcome of the parent trial was pain intensity, measured as a composite pain intensity score [mean of minimum, maximum, and average pain intensity during the last seven days on an 11-point NRS (0–10, anchors: “no pain at all”–“unbearable pain”)] (Carvalho et al., 2016) and recorded by the patients in a patient diary at baseline (day 0), day 11, and day 21 after randomization. A two-point reduction in NRS scores in chronic pain is considered a minimal clinically important difference (MCID; Farrar et al., 2001).

Secondary outcomes included both subjective and objective measures of pain-related disability. The Oswestry Disability Index (ODI) assesses perceived disability across 10 predefined domains of activities of daily living (i.e., personal care, lifting, sitting, standing, sleeping, sexual function, social life, and traveling), thus detecting everyday functional limitations due to CBP (Fairbank et al., 1980). A six to ten point reduction in ODI scores is considered minimally clinically important (Hung et al., 2018). In addition to the ODI, the Patient-Specific Functional Scale (PSFS) was used to identify and quantify functional impairments in activities critical to the individual patient that might not be covered by other standardized questionnaires (Stratford et al., 2009). A reduction of 1.4 points on the PSFS in chronic back pain patients is considered clinically meaningful (Maughan and Lewis, 2010). Objective measures of pain-related disability comprised the range and velocity of spinal motion and the Back Performance Scale (BPS), providing quantifiable data on physical function with a high test-retest reliability (Strand et al., 2002). Hereby, the velocity of motion (VoM) and range of motion (RoM) were recorded by the Epionics SPINE system (Epionics Medical GmbH; Epionics SPINE, Berlin, Germany; Consmüller et al., 2012; Taylor et al., 2010). This system consists of two sensors attached to the right and left Spina Iliaca Posterior Superior and running parallel to the lumbar spine, measuring the surface-bending movements. With the help of acceleration sensors on both ends, they also measured the direction in relation to gravity. The Back Performance Scale provides a standardized protocol in which a blinded experimenter rates five predefined mobility-related activities (i.e., sock test, pick-up test, roll-up test, lift test, and fingertip-to-floor distance), with higher scores indicating a more impaired physical performance (Consmüller et al., 2012; Vaisy et al., 2015). We employed the Veterans RAND 12-Item Health Survey (VR-12) to assess health-related quality of life to quantify a physical and mental component score (VR-12-PCS and VR-12-MCS, respectively). This enables the identification of functional limitations and deficits in wellbeing associated with chronic pain (Selim et al., 2009).

Potential predictors of OLP efficacy comprised self-reported measures of depression, anxiety, and stress, assessed using the Depression Anxiety Stress Scales (DASS; Lovibond and Lovibond, 1995). Pain-related catastrophizing was evaluated using the Pain Catastrophizing Scale (PCS), a 13-item scale measuring the extent of catastrophic thinking triggered by pain (Sullivan et al., 1995a). Exploratory outcomes focused on treatment credibility and expectancy within the OLP+TAU group, which we assessed using the Credibility/Expectancy Questionnaire, a 6-item, easy-to-administer scale used in clinical trials to measure treatment expectancy and credibility.

In this exploratory analysis, we focused primarily on pain-related disability in CBP and its impact on quality of life, as measured by objective measures of spinal mobility (VoM, RoM, and BPS), perceived disability in activities of daily living (ODI, PSFS, BPS), and health-related quality of life (VR-12 PCS and MCS).

### Statistical analysis

Statistical analyses were performed using the software R (The R Project for Statistical Computing, Version 3.4.1, The R Foundation, https://www.r-project.org/) and standardized statistical packages of RStudio (RStudio 2023: Integrated Development Environment for R. Posit Software, PBC, Boston, MA, USA, http://www.rstudio.com/). The significance level was set at p <0.05. Outcome measures included pain-related disability (ODI, PSFS, BPS), quality of life (VR-12 PCS and MCS), and objective measures of spinal mobility (VoM, RoM). Pairwise Pearson correlations were calculated to examine the relationship between pain intensity, pain-related disability (ODI, PSFS), motion characteristics (range and velocity of motion), and the scores of behavioral scales (PCS, DASS). In the light of the correlation analysis, the post-treatment time points (day 21) for all disability measures were tested for differences between factors (OLP+TAU vs. TAU) using a generalized linear model (GLM). The interaction between PCS and treatment was included as a predictor in the model. Pretreatment values were included in the model as a covariate. Separate GLMs, including Body Mass Index (BMI) as a covariate, were performed as sensitivity analyses (see Supplementary material). Estimated parameters are given in mean ± standard error.

## Results

### Participant characteristics

The statistical analyses included 122 patients with chronic back pain (CBP) who completed the trial (OLP+TAU: *N* = 63, TAU: *N* = 59). The OLP+TAU group differed significantly from the TAU group only in terms of body mass index (see Table 1). Detailed group characteristics were reported in the publication of the parental trial (see Kleine-Borgmann et al., 2019 for details).

**Table 1:** Patient characteristics.

**Characteristic**	**TAU group *N* = 59**	**OLP+TAU group *N* = 63**	***p***
**Demography**		
Male	18 (30.5)	28 (44.4)	0.161
Age (in years)	58.37 (13.97)	60.29 (15.15)	0.471
Education (in years)	9.25 (2.51)	8.63 (2.42)	0.371
Body-Mass-Index (in kg/m^2^)	25.72 (5.08)	28.19 (5.37)	0.010
Severity of chronic pain (Von Korff Grade, %; Von Korff et al., 1992)			0.148
Grade I, low disability-low intensity	12 (38.7)	13 (34.2)	
Grade II, low disability-high intensity	0 (0)	0 (0)	
Grade III, high disability-moderately limiting	8 (25.8)	4 (10.5)	
Grade IV, high disability-severely limiting	11 (35.5)	21 (55.3)	
Composite pain intensity score (NRS 0–10)	4.91 (1.97)	5.25 (1.95)	0.341
**Spinal mobility**		
Range of spinal motion (z-transformed, mean, SD)	0.10 (0.72)	−0.08 (0.69)	0.161
Velocity of spinal motion (z-transformed, mean, SD)	−0.05 (0.72)	−0.07 (0.83)	0.876
**Disability**		
Oswestry disability index (in %)	30.17 (12.86)	29.40 (13.12)	0.745
Patient specific functional scale	3.84 (2.04)	4.18 (2.04)	0.359
Back performance scale	4.19 (3.57)	4.70 (3.71)	0.444
**Quality of life**		
VR-12 physical component score	34.33 (10.43)	34.31 (9.75)	0.994
VR-12 mental component score	47.07 (12.18)	49.87 (10.62)	0.180
**Psychological factors**		
Pain catastrophizing scale (mean, SD)	19.30 (10.26)	19.14 (10.70)	0.935
DASS depression score	4.79 (4.29)	4.65 (4.60)	0.865
DASS anxiety score	3.61 (3.66)	4.10 (4.40)	0.519
DASS stress score	7.43 (4.57)	7.03 (5.39)	0.662
CEQ credibility subscale	-	−0.0286(2.58)	-
CEQ expectancy subscale	-	0.0213(2.65)	-

TAU, treatment-as-usual; OLP, open-label placebo; NRS, numeric rating scale; SD, standard deviation; DASS, Depression Anxiety Stress Scale; VR-12, Veterans RAND 12-Item Health Survey; CEQ, Credibility Expectancy Questionnaire. Data given in mean ± standard deviation unless stated otherwise.

### Baseline predictors

We conducted separate GLMs to explore how baseline levels of depression, anxiety, stress, and pain-related catastrophizing predict group-dependent changes in (semi-)objective outcomes such as disability and spinal motion. Additionally, we investigated how treatment credibility and expectancy influenced these outcomes in the OLP-treated group. The analyses revealed that only the model, including pain catastrophizing (PCS), produced significant findings across multiple outcomes, such as velocity of motion (VoM), pain-related disability (ODI), and quality of life (VR-12).

### Pain catastrophizing and spinal mobility

The effect of PCS on velocity of motion on day 21 was negative [estimate = −0.028 ± 0.010, t(104) = −2.737, *p* = 0.007] with a small-to-moderate effect size (*d* = 0.27). This indicates that improved spinal mobility due to OLP+TAU treatment was associated with lower pain catastrophizing at baseline. The strongest predictor of velocity of motion following OLP treatment was the baseline velocity of motion score, reflecting that the initial velocity of motion is a crucial determinant of subsequent outcomes [estimate = 0.912 ± 0.069, t(104) = 13.267, *p* < 0.001]. In contrast, baseline PCS did not influence the effect of OLP treatment on range of motion [estimate = −0.008 ± 0.009, t(104) = −0.862, *p* = 0.391, *d* = −0.085).

### Pain catastrophizing and pain-related disability

Patients with higher PCS scores at baseline who were allocated to the OLP+TAU group demonstrated a trend toward a lower subjective functional disability on day 21 [estimate = −0.248 ± 0.149, t(105) = −1.666, *p* = 0.099, *d* = 0.16]. The GLM analysis also showed a significant positive main effect of baseline PCS on ODI scores across groups [estimate = 0.386 ± 0.120, t(105) = −3.221, *p* = 0.002] with a small-to-moderate effect size (*d* = 0.31). That is, higher levels of pain catastrophizing are associated with higher subjective functional disability, irrespective of the administered treatment. Once more, the baseline scores were the strongest predictor of the scores at day 21 (estimate = 0.637 ± 0.073, *p* < 0.001). Baseline PCS scores did not influence PSFS or BPS significantly on day 21.

### Pain catastrophizing and quality of life

**Figure 1:**
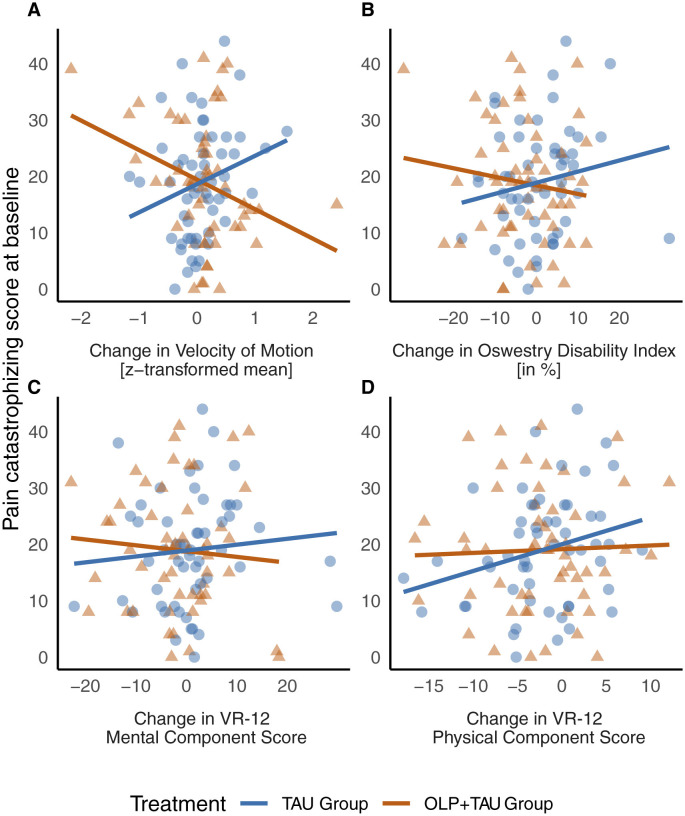
**(A–D)** Interaction effect between group (OLP+TAU vs. OLP) and pain catastrophizing on different measures of disability. Illustrated are changes from day 21 to baseline in the velocity of spinal mobility (reflected as z-transformed scores, **(A)**, disability (measured by Oswestry Disability Index, **(B)**, mental, and physical health (measured by Veteran RAND-12 Mental, **(C)**, and Physical Composite Scores, **(D)**. OLP, open-label placebo; TAU, treatment-as-usual.

PCS scores did not have a significant impact on OLP efficacy in terms of mental or physical health status on day 21 compared to TAU. However, higher PCS scores at baseline were associated with lower physical health outcomes [estimate of −0.184 ± 0.079, t(103) = −2.310, *p* = 0.023] and mental health outcomes [estimate = −0.262 ± 0.113, t(103) = −2.325, *p* = 0.022] independent of the treatment allocation. The effect sizes were small (*d* = 0.22 and *d* = 0.23, respectively). Again, baseline scores were the strongest predictor of follow-up scores [estimate = 0.860 ± 0.058, t(103) = 9.079, *p* < 0.001 and estimate = 0.637 ± 0.070, t(103) = −2.310, *p* < 0.001; Figure 1].

## Discussion

This study presents the findings of a secondary analysis of a large randomized controlled trial that involved 122 patients suffering from chronic back pain, which evaluated the efficacy of adding a 3-week open-label placebo (OLP) treatment to treatment-as-usual (TAU) for chronic back pain. The original study demonstrated that combining OLP with standard treatment significantly improved subjective rather than objective outcomes. In particular, OLP+TAU reduced pain intensity, subjective reports of functional disability, and symptoms of depression. However, there was no effect on objective spinal mobility, i.e., range and velocity of motion (Kleine-Borgmann et al., 2019). This exploratory analysis aimed to identify psychological factors contributing to OLP efficacy on (semi-)objective outcomes such as spinal mobility, pain-related disability, and quality of life.

Across different psychological baseline variables, we identified pain catastrophizing, assessed by the Pain Catastrophizing Scale (Sullivan et al., 1995a), as a significant predictor of OLP-associated changes in spinal velocity of motion and functional disability, i.e., ODI. Specifically, patients with lower levels of pain catastrophizing at baseline, who were administered OLP in addition to TAU, exhibited enhanced spinal mobility in terms of movement velocity by day 21 compared to those who received TAU alone. Conversely, higher levels of pain catastrophizing at baseline were linked to improved functional disability, as indicated by lower ODI scores at day 21 in OLP-treated patients. Unrelated to the treatment group, higher pain catastrophizing scores were associated with lower physical and mental health scores (i.e., VR-12 PCS and MCS subscales), aligning with clinical research evidence.

Even with standard treatment for chronic pain, research has consistently shown that higher levels of pain catastrophizing are associated with maladaptive coping strategies leading to persistent disability, psychological distress, and increased healthcare utilization (Simic et al., 2024). Pain catastrophizing is a maladaptive cognitive process characterized by exaggerated pessimistic predictions of future events and pain-related emotions, often involving magnification of pain perception, rumination on pain-related distress, and a sense of helplessness (Quartana et al., 2009). Individuals who engage in catastrophizing may feel overwhelmed, believing that they are unable to control or manage their pain effectively. This cognitive distortion typically includes components of hopelessness and helplessness, both of which can undermine an individual's sense of self-efficacy and autonomy in coping with pain (Petrini and Arendt-Nielsen, 2020). That is why pain catastrophizing has even been reframed as an unsuccessful problem-solving strategy (Eccleston and Crombez, 2007). Interestingly, OLP efficacy was linked to opposite psychological factors, such as patient self-determination and self-efficacy (Kaptchuk and Miller, 2018), which might explain why OLP treatment in patients with low PCS scores was associated with an increase in the velocity of spinal motion.

Our results are consistent with those previously reported by Ballou et al. (2022), who demonstrated that PCS modulates the effect of OLP treatments on visceral pain. In a previous 6-week RCT exploring placebo effects in irritable bowel syndrome (IBS), researchers examined psychological predictors influencing responses to OLP vs. double-blind placebo (DBP) and no treatment control. The study showed that pain catastrophizing and visceral sensitivity were indicators of response to OLP. Specifically, high visceral sensitivity combined with low pain catastrophizing was linked to greater improvement following OLP treatment. These predictors did not significantly affect the response to DBP, suggesting different mechanisms of action underlying OLP and DBP interventions.

The mechanisms underlying OLP efficacy remain largely unclear. Current hypotheses propose that OLPs might share specific biochemical, psychological, and neuroimaging characteristics with deceptive placebos, particularly in the domain of placebo analgesia (Buergler et al., 2023; Schaefer et al., 2022). Additionally, positive cognitions and an open-minded attitude have been proposed as critical predictors of positive responses to OLPs (Haas et al., 2022). Individual patient engagement in actively assessing and modifying dysfunctional thoughts, akin to techniques used in cognitive behavioral therapy, may be necessary for OLPs to work (Ballou et al., 2022). This assumption is further reinforced by the observation that OLPs may appear paradoxical to patients, necessitating a degree of cognitive flexibility. This requirement may be diminished in individuals with high pain catastrophizing, where dysfunctional cognitive processes contribute to a narrowed focus on pain stimuli (Simic et al., 2024). These patients might be less likely to believe in their ability to positively influence their pain experience, reducing their self-efficacy and the potential benefits of OLPs.

Our findings are less clear regarding functional disability, represented by ODI scores, which indicated that patients with higher PCS at baseline who received OLP treatment exhibited improved disability (i.e., lower ODI scores) on day 21. One explanation of this divergence may lie in the fear-avoidance model of pain, which involves reduced physical activity and increased focus on pain due to the fear of pain highly correlated with pain catastrophizing (Asmundson et al., 1999; Sullivan et al., 2004; Vlaeyen and Linton, 2000). Although higher pain catastrophizing scores may have significantly impaired the efficacy of OLP in a highly consciously perceived movement task, such as the examination of velocity and range of motion due to fear of pain, this effect might have been less pronounced for subjective reports of impairment in daily activities over time. On the contrary, patients with higher levels of catastrophizing may have been more reflective and interpreted subtle positive changes as evidence of the treatment's benefit, even without objective evidence to support this improvement. Therefore, future studies should assess the temporal dynamics of expectations throughout the therapy to better understand their role in shaping patient-reported treatment outcomes.

No interaction effect of PCS with OLP treatment was observed regarding range of motion, back performance scale, and patient-specific functional scale scores. Beyond the methodological concerns raised by Suter et al. (2020), which highlighted moderate to low consistency in the range of motion measurements, we hypothesize that this discrepancy may be partially attributable to the brief duration of OLP treatment. This limited treatment period may be inadequate for improving more anatomically dependent spinal mobility measures, such as range of motion. Therefore, short-term interventions may have been insufficient to capture the gradual and cumulative benefits of OLP in the context of limitations in anatomical changes associated with chronic pain. This secondary analysis supports the concept that subjective outcomes are more responsive to OLPs than objective measures, with psychological factors, such as pain catastrophizing, playing a significant role in their treatment efficacy.

Some limitations must be considered when interpreting these results. To assess pain catastrophizing, we used the validated Pain Catastrophizing Scale, which measures the trait of pain catastrophizing at baseline (Sullivan et al., 1995b). However, the concept of pain catastrophizing and its predictive role in pain-related outcomes is currently the subject of active debate (Quartana et al., 2009). Recent findings from experimental research have introduced a situational component to the concept of pain catastrophizing, suggesting a more dynamic nature of catastrophizing cognitions (Edwards et al., 2005). Furthermore, some argue that tools for assessing pain catastrophizing focus more on pain-related worrying or distress rather than catastrophizing itself, posing a challenge for clinical or experimental research (Petrini and Arendt-Nielsen, 2020). Furthermore, the potential underlying mechanisms of our treatment have been discussed in terms of constructs such as self-efficacy and cognitive flexibility, which we did not directly measure in our experiment. Future studies should include direct measures of these parameters to confirm these hypothesized interpretations.

In conclusion, our secondary analysis of a large randomized controlled trial involving patients with CBP identified pain catastrophizing, measured by the Pain Catastrophizing Scale, as a significant predictor of OLP effects. Patients with lower catastrophizing showed improved spinal velocity of motion, while those with higher levels of catastrophizing experienced greater improvements in functional disability. The results align with the concept of fear-avoidance of pain and highlight the relevance of psychological factors for OLP efficacy in CBP. In line with previous research, our analysis points to distinct mechanisms of OLP for subjective and objective outcomes. Future research should focus on personalized OLP treatment for chronic pain conditions by understanding the psychological factors involved, including their temporal dynamics.

## Data Availability

The datasets presented in this article are not readily available because participant consent for data sharing was not obtained at the time of acquisition. Additionally, the underlying health data contains sensitive, personally identifiable information, which necessitates strict confidentiality to protect participant privacy. Consequently, sharing or redistributing this data via open repositories would be incompatible with both ethical standards and data protection regulations. Requests to access the datasets should be directed to julian.kleine-borgmann@uk-essen.de.

## References

[B1] Asmundson G. J. G. Norton P. J. Norton G. R. (1999). Beyond pain: the role of fear and avoidance in chronicity. Clin. Psychol. Rev. 19, 97–119. 10.1016/S0272-7358(98)00034-8s 9987586

[B2] Ballou S. Haas J. W. Iturrino J. Nee J. Kirsch I. Rangan V.. (2022). Psychological predictors of response to open-label versus double-blind placebo in a randomized controlled trial in irritable bowel syndrome. Psychosom. Med. 84:738. 10.1097/PSY.0000000000001078 35412513 PMC9271597

[B3] Benedetti F. Shaibani A. Arduino C. Thoen W. (2023). Open-label nondeceptive placebo analgesia is blocked by the opioid antagonist naloxone. Pain 164, 984–990. 10.1097/j.pain.0000000000002791 36165878

[B4] Birbara C. A. Puopolo A. D. Munoz D. R. Sheldon E. A. Mangione A. Bohidar N. R.. (2003). Treatment of chronic low back pain with etoricoxib, a new cyclo-oxygenase-2 selective inhibitor: improvement in pain and disability - a randomized, placebo-controlled, 3-month trial. J. Pain 4, 307–315. 10.1016/S1526-5900(03)00633-3 14622687

[B5] Blease C. R. Bernstein M. H. Locher C. (2020). Open-label placebo clinical trials: is it the rationale, the interaction or the pill? BMJ Evid. Based Med. 25, 159–165. 10.1136/bmjebm-2019-111209 31243047 PMC6930978

[B6] Buergler S. Sezer D. Gaab J. Locher C. (2023). The roles of expectation, comparator, administration route, and population in open-label placebo effects: a network meta-analysis. Sci. Rep. 13, 1–12. 10.1038/s41598-023-39123-4 37481686 PMC10363169

[B7] Carvalho C. Caetano J. M. Cunha L. Rebouta P. Kaptchuk T. J. Kirsch I. (2016). Open-label placebo treatment in chronic low back pain: a randomized controlled trial. Pain 157:2766. 10.1097/j.pain.0000000000000700 27755279 PMC5113234

[B8] Consmüller T. Rohlmann A. Weinland D. Druschel C. Duda G. N. Taylor W. R. (2012). Comparative evaluation of a novel measurement tool to assess lumbar spine posture and range of motion. Eur. Spine J. 21, 2170–2180. 10.1007/s00586-012-2312-1 22543411 PMC3481097

[B9] Eccleston C. Crombez G. (2007). Worry and chronic pain: a misdirected problem solving model. Pain 132, 233–236. 10.1016/j.pain.2007.09.014 17961924

[B10] Edwards R. R. Campbell C. M. Fillingim R. B. (2005). Catastrophizing and experimental pain sensitivity: only *in vivo* reports of catastrophic cognitions correlate with pain responses. J. Pain 6, 338–339. 10.1016/j.jpain.2005.02.013 15890636

[B11] Ehde D. M. Dillworth T. M. Turner J. A. (2014). Cognitive-behavioral therapy for individuals with chronic pain: efficacy, innovations, and directions for research. Am. Psychol. 69, 153–166. 10.1037/a0035747 24547801

[B12] Enck P. Bingel U. Schedlowski M. Rief W. (2013). The placebo response in medicine: minimize, maximize or personalize? Nat. Rev. Drug Discov. 12, 191–204. 10.1038/nrd3923 23449306

[B13] Fairbank J. C. Couper J. Davies J. B. O'Brien J. P. (1980). The Oswestry low back pain disability questionnaire. Physiotherapy 66, 271–273. 10.1037/t04205-000 6450426

[B14] Farrar J. T. Young J. P. LaMoreaux L. Werth J. L. Poole R. M. (2001). Clinical importance of changes in chronic pain intensity measured on an 11-point numerical pain rating scale. Pain 94, 149–158. 10.1016/S0304-3959(01)00349-9 11690728

[B15] Ferreira M. L. De Luca K. Haile L. M. Steinmetz J. D. Culbreth G. T. Cross M.. (2023). Global, regional, and national burden of low back pain, 1990–2020, its attributable risk factors, and projections to 2050: a systematic analysis of the Global Burden of Disease Study 2021. Lancet Rheumatol. 5, e316–e329. 10.1016/S2665-9913(23)00098-X 37273833 PMC10234592

[B16] Haas J. W. Ongaro G. Jacobson E. Conboy L. A. Nee J. Iturrino J.. (2022). Patients' experiences treated with open-label placebo versus double-blind placebo: a mixed methods qualitative study. BMC Psychol. 10:20. 10.1186/s40359-022-00731-w 35120572 PMC8815135

[B17] Hung M. Saltzman C. L. Kendall R. Bounsanga J. Voss M. W. Lawrence B.. (2018). What Are the MCIDs for PROMIS, NDI, and ODI instruments among patients with spinal conditions? Clin. Orthop. Relat. Res. 476, 2027–2036. 10.1097/CORR.0000000000000419 30179950 PMC6259866

[B18] Jensen K. B. Kaptchuk T. J. Chen X. Kirsch I. Ingvar M. Gollub R. L.. (2015). A neural mechanism for nonconscious activation of conditioned placebo and nocebo responses. Cereb Cortex. 25, 3903–3910. 10.1093/cercor/bhu275 25452576 PMC4585522

[B19] Kaptchuk T. J. Hemond C. C. Miller F. G. (2020). Placebos in chronic pain: evidence, theory, ethics, and use in clinical practice. BMJ 370:m1668. 10.1136/bmj.m1668 32690477

[B20] Kaptchuk T. J. Miller F. G. (2015). Placebo effects in medicine. N. Engl. J. Med. 373, 8–9. 10.1056/NEJMp1504023 26132938

[B21] Kaptchuk T. J. Miller F. G. (2018). Open label placebo: can honestly prescribed placebos evoke meaningful therapeutic benefits? BMJ 363:k3889. 10.1136/bmj.k3889 30279235 PMC6889847

[B22] Kleine-Borgmann J. Dietz T. N. Schmidt K. Bingel U. (2023). No long-term effects after a 3-week open-label placebo treatment for chronic low back pain: a 3-year follow-up of a randomized controlled trial. Pain 164, 645–652. 10.1097/j.pain.0000000000002752 35947884 PMC9916047

[B23] Kleine-Borgmann J. Schmidt K. Hellmann A. Bingel U. (2019). Effects of open-label placebo on pain, functional disability, and spine mobility in patients with chronic back pain: a randomized controlled trial. Pain 160, 2891–2897. 10.1097/j.pain.0000000000001683 31479068

[B24] Lovibond P. F. Lovibond S. H. (1995). The structure of negative emotional states: comparison of the depression anxiety stress scales (DASS) with the beck depression and anxiety inventories. Behav. Res. Ther. 33, 335–343. 10.1016/0005-7967(94)00075-U 7726811

[B25] Machado G. C. Maher C. G. Ferreira P. H. Pinheiro M. B. Lin C. W. C. Day R. O.. (2015). Efficacy and safety of paracetamol for spinal pain and osteoarthritis: systematic review and meta-analysis of randomised placebo controlled trials. BMJ 350:h1225. 10.1136/bmj.h1225 25828856 PMC4381278

[B26] Maughan E. F. Lewis J. S. (2010). Outcome measures in chronic low back pain. Eur. Spine J. 19, 1484–1494. 10.1007/s00586-010-1353-6 20397032 PMC2989277

[B27] Meucci R. D. Fassa A. G. Xavier Faria N. M. (2015). Prevalence of chronic low back pain: systematic review. Rev. Saude Publica. 49:1. 10.1590/S0034-8910.2015049005874 26487293 PMC4603263

[B28] Petrie K. J. Rief W. (2019). Psychobiological mechanisms of placebo and nocebo effects: pathways to improve treatments and reduce side effects. Annu. Rev. Psychol. 70, 599–625. 10.1146/annurev-psych-010418-102907 30110575

[B29] Petrini L. Arendt-Nielsen L. (2020). Understanding pain catastrophizing: putting pieces together. Front. Psychol. 11:603420. 10.3389/fpsyg.2020.603420 33391121 PMC7772183

[B30] Quartana P. J. Campbell C. M. Edwards R. R. (2009). Pain catastrophizing: a critical review. Expert Rev. Neurother. 9, 745–758. 10.1586/ern.09.34 19402782 PMC2696024

[B31] Rossettini G. Campaci F. Bialosky J. Huysmans E. Vase L. Carlino E. (2023). The biology of placebo and nocebo effects on experimental and chronic pain: state of the art. J Clin Med. 12:4113. 10.3390/jcm12124113 37373806 PMC10299252

[B32] Schaefer M. Kühnel A. Schweitzer F. Enge S. Gärtner M. (2022). Neural underpinnings of open-label placebo effects in emotional distress. Neuropsychopharmacology 48, 560–566. 10.1038/s41386-022-01501-3 36456814 PMC9852452

[B33] Selim A. J. Rogers W. Fleishman J. A. Qian S. X. Fincke B. G. Rothendler J. A.. (2009). Updated U.S. population standard for the veterans RAND 12-item health survey (VR-12). Qual. Life Res. 18, 43–52. 10.1007/s11136-008-9418-2 19051059

[B34] Simic K. Savic B. Knezevic N. N. (2024). Pain catastrophizing: how far have we come. Neurol. Int. 16, 483–501. 10.3390/neurolint16030036 38804476 PMC11130925

[B35] Skyt I. Vase L. (2016). Placebo effects in chronic pain conditions. Can placebo components enhance the efficacy of active treatments? Tidsskrift for Forskning i Sygdom Og Samfund 12, 63–88. 10.7146/tfss.v12i23.22895

[B36] Strand L. I. Moe-Nilssen R. Ljunggren A. E. (2002). Back performance scale for the assessment of mobility-related activities in people with back pain. Phys. Ther. 82, 1213–1223. 10.1093/ptj/82.12.1213 12444880

[B37] Stratford P. Gill C. Westaway M. Binkley J. (2009). Assessing disability and change on individual patients: a report of a patient specific measure. 47, 258–263. 10.3138/ptc.47.4.258

[B38] Sullivan M. J. L. Bishop S. R. Pivik J. (1995a). The Pain Catastrophizing Scale User Manual. APA PsycTests Database. 10.1037/t01304-000

[B39] Sullivan M. J. L. Bishop S. R. Pivik J. (1995b). The pain catastrophizing scale: development and validation. Psychol. Assess. 7, 524–532. 10.1037/1040-3590.7.4.524

[B40] Sullivan M. J. L. Thorn B. Rodgers W. Ward L. C. (2004). Path model of psychological antecedents to pain experience: experimental and clinical findings. Clin. J. Pain. 20, 164–173. 10.1097/00002508-200405000-00006 15100592

[B41] Suter M. Eichelberger P. Frangi J. Simonet E. Baur H. Schmid S. (2020). Measuring lumbar back motion during functional activities using a portable strain gauge sensor-based system: a comparative evaluation and reliability study. J Biomech. 100:109593. 10.1016/j.jbiomech.2019.109593 31898974

[B42] Taylor W. R. Consmüller T. Rohlmann A. (2010). A novel system for the dynamic assessment of back shape. Med. Eng. Phys. 32, 1080–1083. 10.1016/j.medengphy.2010.07.011 20708957

[B43] Vaisy M. Gizzi L. Petzke F. Consmüller T. Pfingsten M. Falla D. (2015). Measurement of lumbar spine functional movement in low back pain. Clin. J. Pain. 31, 876–885. 10.1097/AJP.0000000000000190 25503596

[B44] van der Gaag W. H. Roelofs P. D. D. M. Enthoven W. T. M. van Tulder M. W. Koes B. W. (2020). Non-steroidal anti-inflammatory drugs for acute low back pain. Cochrane Database Syst. Rev. 4:CD013581. 10.1002/14651858.CD013581 32297973 PMC7161726

[B45] Vlaeyen J. W. S. Linton S. J. (2000). Fear-avoidance and its consequences in chronic musculoskeletal pain: a state of the art. Pain 85, 317–332. 10.1016/S0304-3959(99)00242-0 10781906

[B46] Von Korff M. Ormel J. Keefe F. J. Dworkin S. F. (1992). Grading the severity of chronic pain. Pain 50, 133–149. 10.1016/0304-3959(92)90154-4 1408309

[B47] von Wernsdorff M. Loef M. Tuschen-Caffier B. Schmidt S. (2021). Effects of open-label placebos in clinical trials: a systematic review and meta-analysis. Sci. Rep. 11:3855. 10.1038/s41598-021-83148-6 33594150 PMC7887232

