# Supplementary material for: From catastrophizing to catalyzing: does pain catastrophizing modulate the beneficial impact of open-label placebos for chronic low back pain? A secondary analysis
Source: Front Psychol. 2025 Jun 25;16:1522634. doi: 10.3389/fpsyg.2025.1522634 (PMC12239988; doi:10.3389/fpsyg.2025.1522634)
Supplement: Supplementary file 1 [file Data_Sheet_1.docx]

Supplement
Result Tables

**Table S1 – Result table: Generalized linear model (GLM), velocity of spinal motion.**

| GLM for VoM  Groups: Treatment as usual group (TAU),  Open-label-placebo group (OLP+TAU) Reference group: TAU | Estimates (b) | df | t | p | d | η^2^ |
| --- | --- | --- | --- | --- | --- | --- |
| OLP+TAU group | 0.56 ± 0.22 | 104 | 2.56 | 0.012 | 0.25 | <0.01 |
| Baseline score of Pain Catastrophizing Scale (PCS) | 0.01 ± 0.01 | 104 | 1.33 | 0.187 | 0.13 | <0.01 |
| Baseline VoM | 0.91 ± 0.07 | 104 | 13.27 | <0.001 | 1.30 | 0.62 |
| Interaction OLP*Baseline score of PCS | -0.03 ± 0.01 | 104 | -2.74 | 0.007 | -0.27 | 0.03 |

Presented are mean, unstandardized GLM estimates (b) ± standard error of group differences, Cohen’s d (d) and partial η^2^ (R^2^ = 0.79). VoM: Velocity of Motion, OLP: Open-Label Placebo, TAU: Treatment-as-usual, PCS: Pain Catastrophizing Scale.

**Table S2– Result table: Generalized linear model (GLM), range of spinal motion.**

| GLM for RoM  Groups: Treatment as usual group (TAU),  Open-label-placebo group (OLP+TAU) Reference group: TAU | Estimates (b) | df | t | p | d | η^2^ |
| --- | --- | --- | --- | --- | --- | --- |
| OLP+TAU group | 0.17 ± 0.19 | 104 | 0.89 | 0.377 | 0.09 | <0.01 |
| Baseline score of Pain Catastrophizing Scale (PCS) | 0.00 ± 0.01 | 104 | 0.354 | 0.724 | 0.04 | <0.01 |
| Baseline score of RoM | 0.83 ± 0.07 | 104 | 11.69 | <0.001 | 1.15 | 0.56 |
| Interaction OLP*Baseline score of PCS | -0.01 ± 0.01 | 104 | -0.86 | 0.391 | -0.09 | <0.01 |

Presented are mean, unstandardized GLM estimates (b) ± standard error of group differences, Cohen’s d (d) and partial η^2^ (R^2^ = 0.39). RoM: Range of Motion, OLP: Open-Label Placebo, TAU: Treatment-as-usual, PCS: Pain Catastrophizing Scale.

**Table S3 – Result table: Generalized linear model (GLM), Oswestry Disability Index.**

| GLM for ODI  Groups: Treatment as usual group (TAU),  Open-label-placebo group (OLP+TAU) Reference group: TAU | Estimates (b) | df | t | p | d | η^2^ |
| --- | --- | --- | --- | --- | --- | --- |
| OLP+TAU group | 0.76 ± 3.20 | 105 | 0.24 | 0.813 | 0.02 | 0.02 |
| Baseline score of Pain Catastrophizing Scale (PCS) | 0.39 ± 0.12 | 105 | 3.22 | 0.002 | 0.31 | 0.03 |
| Baseline score of Oswestry Disability Index | 0.64 ± 0.07 | 105 | 8.73 | <0.001 | 0.85 | 0.28 |
| Interaction OLP*Baseline score of PCS | -0.25 ± 0.15 | 105 | -0.17 | 0.099 | -0.16 | 0.01 |

Presented are mean, unstandardized GLM estimates (b) ± standard error of group differences, Cohen’s d (d) and partial η^2^ (R^2^ = 0.12). ODI: Oswestry Disability Index, OLP: Open-Label Placebo, TAU: Treatment-as-usual, PCS: Pain Catastrophizing Scale.

**Table S4 – Result table: Generalized linear model (GLM), Patient Specific Functional Scale.**

| GLM for PSFS  Groups: Treatment as usual group (TAU),  Open-label-placebo group (OLP+TAU) Reference group: TAU | Estimates (b) | df | t | p | d | η^2^ |
| --- | --- | --- | --- | --- | --- | --- |
| OLP+TAU group | 0.58 ± 0.77 | 105 | 0.76 | 0.452 | 0.54 | 0.02 |
| Baseline score of Pain Catastrophizing Scale (PCS) | - 0.02 ± 0.03 | 105 | - 0.60 | 0.548 | -0.05 | 0.01 |
| Baseline score of PSFS | 0.40 ± 0.09 | 105 | 4.32 | <0.001 | 0.43 | 0.15 |
| Interaction OLP*Baseline score of PCS | 0.00 ± 0.04 | 105 | 0.07 | 0.941 | -0.01 | <0.01 |

Presented are mean, unstandardized GLM estimates (b) ± standard error of group differences, Cohen’s d (d) and partial η^2^ (R^2^ = 0.05). PSFS: Patient Specific Functional Scale, OLP: Open-Label Placebo, TAU: Treatment-as-usual, PCS: Pain Catastrophizing Scale.

**Table S5 – Result table: Generalized linear model (GLM), Back Performance Scale.**

| GLM for BPS  Groups: Treatment as usual group (TAU),  Open-label-placebo group (OLP+TAU) Reference group: TAU | Estimates (b) | df | t | p | d | η^2^ |
| --- | --- | --- | --- | --- | --- | --- |
| OLP+TAU group | 0.00 ± 0.59 | 103 | 0.01 | 0.996 | -0.00 | <0.01 |
| Baseline score of Pain Catastrophizing Scale (PCS) | 0.01 ± 0.02 | 103 | 0.36 | 0.718 | -0.03 | <0.01 |
| Baseline score of BPS | 0.84 ± 0.04 | 103 | 20.40 | <0.001 | 2.01 | 0.77 |
| Interaction OLP*Baseline score of PCS | -0.01 ± 0.03 | 103 | -0.47 | 0.641 | 0.05 | <0.01 |

Presented are mean, unstandardized GLM estimates (b) ± standard error of group differences, Cohen’s d (d) and partial η^2^ (R^2^ = 0.32). BPS: Back Performance Scale, OLP: Open-Label Placebo, TAU: Treatment-as-usual, PCS: Pain Catastrophizing Scale.

**Table S6 – Result table: Generalized linear model (GLM), Veterans Rand 12-item Mental Component Score.**

| GLM for VR-12 MCS  Groups: Treatment as usual group (TAU),  Open-label-placebo group (OLP+TAU) Reference group: TAU | Estimates (b) | df | t | p | d | η^2^ |
| --- | --- | --- | --- | --- | --- | --- |
| OLP+TAU group | -0.60 ± 3.14 | 103 | -0.19 | 0.850 | -0.02 | 0.02 |
| Baseline score of Pain Catastrophizing Scale (PCS) | -0.26 ± 0.11 | 103 | -2.33 | 0.022 | -0.23 | 0.02 |
| Baseline score of VR-12 MCS | 0.64 ± 0.07 | 103 | 9.08 | <0.001 | 0.89 | 0.36 |
| Interaction OLP*Baseline score of PCS | 0.20 ± 0.15 | 103 | 1.40 | 0.164 | 0.14 | 0.01 |

Presented are mean, unstandardized GLM estimates (b) ± standard error of group differences, Cohen’s d (d) and partial η^2^ (R^2^ = 0.10). VR-12 MCS: Veterans Rand 12-item Mental Component Score, OLP: Open-Label Placebo, TAU: Treatment-as-usual, PCS: Pain Catastrophizing Scale.

**Table S7 – Result table: Generalized linear model (GLM), Veterans Rand 12-item Physical Component Score.**

| GLM for VR-12 PCS  Groups: Treatment as usual group (TAU),  Open-label-placebo group (OLP+TAU) Reference group: TAU | Estimates (b) | df | t | p | d | η^2^ |
| --- | --- | --- | --- | --- | --- | --- |
| OLP+TAU group | -1.73 ± 2.30 | 103 | 3.72 | <0.001 | -0.07 | <0.01 |
| Baseline score of Pain Catastrophizing Scale (PCS) | -0.18 ± 0.08 | 103 | -2.31 | 0.023 | -0.22 | 0.01 |
| Baseline score of VR-12 PCS | 0.86 ± 0.06 | 103 | 14.87 | <0.001 | 1.47 | 0.58 |
| Interaction OLP*Baseline score of PCS | 0.11 ± 0.11 | 103 | 1.04 | 0.302 | 0.10 | <0.01 |

Presented are mean, unstandardized GLM estimates (b) ± standard error of group differences, Cohen’s d (d) and partial η^2^ (R^2^ = 0.17). VR-12 PCS: Veterans Rand 12-item Physical Component Score, OLP: Open-Label Placebo, TAU: Treatment-as-usual, PCS: Pain Catastrophizing Scale.

Sensitivity Analyses

**Table S8 – Sensitivity analysis: Generalized linear model (GLM), Velocity of Motion**

| GLM for VoM  Groups: Treatment as usual group (TAU),  Open-label-placebo group (OLP+TAU) Reference group: TAU | Estimates (b) | df | t | p | d | η^2^ |
| --- | --- | --- | --- | --- | --- | --- |
| OLP+TAU group | 0.57 ± 0.31 | 108 | 2.55 | 0.013 | 0.07 | <0.01 |
| Baseline score of Pain Catastrophizing Scale (PCS) | 0.01 ± 0.01 | 108 | 1.32 | 0.189 | 0.21 | 0.01 |
| Baseline velocity of spinal motion | 0.91 ± 0.07 | 108 | 13.10 | <0.001 | 2.55 | 0.62 |
| Body Mass Index (kg/m^2^) | -0.01 ± 0.01 | 108 | -0.14 | 0.886 | 0.03 | <0.01 |
| Interaction OLP*Baseline score of PCS | -0.03 ± 0.01 | 108 | -2.73 | 0.007 | 0.54 | 0.06 |

Presented are mean, unstandardized GLM estimates (b) ± standard error of group differences, Cohen’s d and partial η^2^ (R^2^ = 0.69). VoM: Velocity of Motion, OLP: Open-Label Placebo, TAU: Treatment-as-usual, PCS: Pain Catastrophizing Scale.

**Table S9 – Sensitivity analysis: Generalized linear model (GLM), Oswestry Disability Index**

| GLM for ODI  Groups: Treatment as usual group (TAU),  Open-label-placebo group (OLP+TAU) Reference group: TAU | Estimates (b) | df | t | p | d | η^2^ |
| --- | --- | --- | --- | --- | --- | --- |
| OLP+TAU group | -0.72 ± 3.12 | 109 | -0.23 | 0.818 | 0.66 | 0.10 |
| Baseline score of Pain Catastrophizing Scale (PCS) | 0.39 ± 0.12 | 109 | 3.35 | 0.001 | 0.60 | 0.08 |
| Baseline score of Oswestry Disability Index | 0.63± 0.07 | 109 | 8.96 | <0.001 | 1.76 | 0.44 |
| Body Mass Index (kg/m^2^) | 0.42 ± 0.14 | 109 | 3.05 | 0.003 | 0.60 | 0.08 |
| Interaction OLP*Baseline score of PCS | -0.23± 0.14 | 109 | -1.62 | 0.108 | 0.32 | 0.02 |

Presented are mean, unstandardized GLM estimates (b) ± standard error of group differences, Cohen’s d and partial η^2^ (R^2^ = 0.65). ODI: Oswestry Disability Index, OLP: Open-Label Placebo, TAU: Treatment-as-usual, PCS: Pain Catastrophizing Scale.
